# Biomimetic Plant-Root-Inspired Robotic Sensor System

**DOI:** 10.3390/bios14120565

**Published:** 2024-11-22

**Authors:** Margarita Alvira, Alessio Mondini, Gian Luigi Puleo, Islam Bogachan Tahirbegi, Lucia Beccai, Ali Sadeghi, Barbara Mazzolai, Mònica Mir, Josep Samitier

**Affiliations:** 1 Nanobioengineering Group, Institute for Bioengineering of Catalonia (IBEC), Barcelona Institute of Science and Technology (BIST), 12 Baldiri Reixac 15-21, 08028 Barcelona, Spainjsamitier@ibecbarcelona.eu (J.S.); 2 Bioinspired Soft Robotics Laboratory, Istituto Italiano di Tecnologia, 16163 Genova, Italy; alessio.mondini@iit.it (A.M.); barbara.mazzolai@iit.it (B.M.); 3 Soft BioRobotics Perception Lab, Istituto Italiano di Tecnologia, 16163 Genova, Italy; lucia.beccai@iit.it; 4 Department of Electronics and Biomedical Engineering, University of Barcelona, Martí i Franquès 1, 08028 Barcelona, Spain; 5 Centro de Investigación Biomédica en Red de Bioingeniería, Biomateriales y Nanomedicina, Instituto de Salud Carlos III, 08028 Barcelona, Spain

**Keywords:** chemical sensor, biomimetic, pH, potassium, plant roots, tropism, ion-selective electrode (ISE), soil detection, robotics, artificial intelligence

## Abstract

There are many examples in nature in which the ability to detect is combined with decision-making, such as the basic survival instinct of plants and animals to search for food. We can technically translate this innate function via the use of robotics with integrated sensors and artificial intelligence. However, the integration of sensing capabilities into robotics has traditionally been neglected due to the significant associated technical challenges. Inspired by plant-root chemotropism, we present a miniaturized electrochemical array integrated into a robotic tip, embedding a customized micro-potentiometer. The system contains solid-state sensors fitted to the tip of the robotic root to three-dimensionally monitor potassium and pH changes in a moist, soil-like environment, providing an integrated electronic readout. The sensors measure a range of parameters compatible with realistic soil conditions. The sensors’ response can trigger the movement of the robotic root with a control algorithm inspired by the behavior of the plant root that determines the optimal path toward root growth, simulating the decision-making process of a plant. This nature-inspired technology may lead, in the future, to the realization of robotic devices with the potential for monitoring and exploring the soil autonomously.

## 1. Introduction

As sessile organisms, plants depend on their ability to sense and respond to subtle environmental signals, such as water distribution, soil mechanical impedance, wind, nutrients, gravity, temperature, light changes, and chemical presence, for their survival and reproductive success. Behavioral replies to these stimuli, concerning the growth direction of plant structures, are known as tropisms, and they represent one of the most fascinating adaptation aspects in nature [1,2,3]. A formidable example of these adaptation strategies is offered by plant roots, which evolved the ability to both sense several factors in their local environment and use this information to drive changes in growth direction to optimize underground nutrient and water exploitation [4]. During this central task, roots display an extremely accurate chemical sensing capability, used mainly in searching for nutrient molecules and ions [5] such as nitrate (NO_3_^−^) and hydrogen phosphate (HPO_4_^2−^), as well as oxonium (H_3_O^+^) to balance the soil pH [6], but also related to the avoidance of poisons and stress resistance into the soil [7].

However, the localization of the plant root’s active center for chemosensitivity is still an open issue in plant physiology [2]. It was reported that, for NO_3_^−^ [8] and HPO_4_^2−^ [9], the root apical cells are more sensitive to chemical concentration gradients, considering their reduced vacuolar dimensions and their consequent reduced homeostasis response [8]. The latter explanation can be extended for K^+^ and for H_3_O^+^, suggesting that the root apical region plays a central role in chemosensitivity [8,10], in addition to other sensing processes related to plant tropism, such as mechanosensing and gravitropic sensing [11].

With this simplified picture, plant-root apexes offer a very interesting model for a sensitive, artificial, and multiresponsive system, able to display chemotropism, intended as the capability to grow in a direction following positive chemical stimuli (i.e., nutrients) and avoiding negative chemical stimuli that are noxious for the plant (i.e., toxins).

Bionanotechnology has focused many efforts on learning from natural morphology, such as the gecko-footpad hairs that inspired a new, reversible wet/dry adhesive [12], and on emulating animal locomotion and behaviors, such as the dog-sized robot developed by Ruppert et al., which learns how to walk from the experiences recorded by the robot [13]. But the complex biology of plants has been less studied in biotechnology, although it offers many interesting mechanisms including chemotropism, among others.

Although great possibilities are offered by robotics and biosensor technologies [14,15], few examples of these combined technologies have been reported. The integration of sensors in robotics is limited mainly to physical sensors, such as two-dimensional and three-dimensional visual sensors [16], force/torque sensors [17], and collision detection sensors [18].

The main example of chemical sensors in robotics is found in gas sensors, also called electronic noses, installed on wheeled or flying robots to analyze a chemical plume trace to detect the source of chemical release. In these papers, the use of well-established, commercially available gas sensors based on non-functionalized metal-oxide-semiconductors [19,20] or commercialized optical detectors to trace a fluorescent dye in water [21] is reported. But these basic, non-functionalized sensors are limited to certain types of samples. On the other hand, functionalized sensors can detect any type of target, and the implementation of this type of sensor opens the possibility of detecting a wide range of diverse analytes, potentiating applications in many different areas.

To the authors’ knowledge, just a couple of examples of functionalized chemical sensors embedded in robotics have been published. The robotic finger for the screening of flavors and additives reported by Ciui et al. is an example [22]. In this system, sensors built into gloves worn on a robotic hand can differentiate spiciness, sourness, and sweetness, via the direct electrochemical detection of capsaicin and ascorbic acid, respectively, and of glucose, which requires sensor functionalization with glucose oxidase and mediator [22]. Robotic fish developed by Ravalli et al. for water quality monitoring in fish farming cages have also been reported. This technology was able to detect the pH in water by means of an electrochemical sensor based on the modification of the surface with a proton-sensitive polyaniline film, with pH changes triggering a change in the robot’s swimming patterns [23].

However, robots combined with biosensor and chemical sensor technology are excellent tools that, when combined, offer many more possibilities and allow the imitation of plant performance, among other behaviors. New applications of bioinformatics engineering, artificial intelligence, and machine learning may emerge through the use of these integrated technologies for environmental monitoring, precision agriculture, precision medicine, and next-generation biomanufacturing.

In this work, we were inspired by the chemotropism of plant roots to develop a new chemical sensor integrated into a robotic root that stimulates the decision-making process by sensing potassium concentration and pH, monitored by integrated multiparameter ion-selective electrodes (ISEs). As a result, the robot reaches out toward the highest concentration of these ions, mimicking the behavior of a plant root in terms of targeting nutrients for growth. This system was developed to robustly map the highest nutrient contents in soil.

The artificial intelligence of this platform, which connects the sensory part with the robot, perceives its environment and reacts to maximize its chances of reaching the highest concentration of nutrients, generating a movement in a specific 3D context to make the robot grow in that direction. The ISE technology uses a simple experimental setup in which the potential of the working electrode is measured against that of the reference electrode under zero-current conditions. The potential is measured by a simple voltmeter with high input impedance. These electrodes and their accompanying electronic circuits, integrated into the robotic system for data acquisition, present an excellent basis for the development of an inexpensive, and ideally disposable, detection system that can be deployed for in situ analysis. However, the current costs and the size of commercially available ISEs do not allow their integration into autonomous detection systems that can be deployed in the field. In this platform, the electrical circuit of the voltmeter was integrated into the robotic circuit to miniaturize the readout and reduce costs.

For the validation of the prototype, a gradient of pH and potassium was developed in a moist, soil-like environment, resembling what is naturally produced in soil. Phytagel^®^ was used to mimic wet soil, providing a controlled ionic environment that allows the testing of robotic behavior within a few minutes. This “artificial soil system” is an agar substitute for plant tissue culture and has been previously used to track plant-root movements [24,25]. This setup allows the recapitulation of wet soil conditions without needing systems for ion extraction and facilitates quick responses from the robot tip. The integration of the ISE sensors of each kind in the artificial tip of a plant-like robot, enabled the development of the robotic root behavior in the presence of a simulated chemical gradient, opening new perspectives into chemotropism analysis and autonomous robotic soil monitoring systems.

This is an innovative approach considering the lack of development of robotics and information and communication technologies (ICTs) for autonomous soil exploration and in situ ground environmental monitoring, compared to laboratory methods of soil analysis. Considering an alternative sensor, this combination of technologies can also be applied to the detection of pesticides/soil toxicity to map environmental problems or to search for components with high added value in soils.

## 2. Materials and Methods

### 2.1. Electrochemical Sensor Array Design, Fabrication, and Materials

An array of pH and potassium ion-selective electrodes (ISEs) was designed on screen-printed electrodes. Each sensor array is composed of three electrodes: two carbon working electrodes (WEs) and one silver/silver chloride reference electrode (RE). The miniaturized screen-printed electrodes were customized and manufactured by DropSens (Llaneras, Spain).

Ionophores (valinomycin and tridecylamine), plasticizer (bis(1-butylpentyl) adipate (BBPA)), lipophilic additive (potassium tetrakis (4-chlorophenyl) borate (KTClPB)), high-molecular-weight poly (vinyl chloride) (PVC), and Phytagel^TM^ were obtained from Sigma Aldrich, Darmstadt, Germany. Tetrahydrofuran (THF), tris(hydroxymethyl)aminomethane (Tris), MgSO_4_, HCl, and KCl were supplied by Panreac. All the reagents were of analytical grade. The epoxy glue used in the sensor wiring and integration was the “DoubleBubble” from Loctite (Düsseldorf, Germany). In all the experiments, twice-distilled water was used.

The carbon WEs were modified with 2 µL of K^+^ or pH ISE cocktail. The pH ISE membrane cocktail was prepared using the following composition: 1.0 wt% tridecylamine (H^+^ ionophore I), 0.5 wt% of KTClPB, 65.5 wt% of BBPA, and 33 wt% high-molecular-weight PVC. The ISE membrane for potassium detection was prepared with a mixture of 2 wt% potassium ionophore valinomycin, 64.8 wt% BBPA, 0.5 wt% KTClPB, and 32.7 wt% PVC. In total, 200 mg of this mixture was dissolved in 2 mL of anhydrous THF. The sensors were integrated in a robotic root tip [26]. The tip was already equipped with four types of sensors (gravity, soil moisture, temperature, and touch) and a bioinspired algorithm to implement tropic behaviors. The root tip position was managed by a three-spring soft-bending mechanism to test the sensors and behavior in air before facing soil. The three pH-K^+^ sensor arrays were inserted in three housings realized at the tip’s external surface. Each sensor was wired and connected with the electronic board through a connector. The external soldering pads and openings necessary to pass the wires were insulated and closed with epoxy glue.

### 2.2. Electronics and Control

Several issues were faced in integrating the ISE sensors in the artificial tip, as well as in conditioning and reading their output signals near the sensors’ site. The type of measurement was potentiometric and a particular configuration of the sensor arrays, with two WEs and a single RE, was considered.

A chemical-sensor-conditioning front-end (named “micro-potentiometer”) was developed for the root tip electronics, which consisted of a board (4 cm in diameter) integrating a microcontroller unit and a communication unit. To integrate the potentiometric measurement system in the apex, a trade-off between its dimensions and final performance was necessary. For each electrode, a high-input-impedance amplifier was used (INA331 from Texas Instruments (Dallas, TX, USA)). The RE was driven using three individual reference voltages (of the same value) supplied with a high-value resistance (10 MΩ) to limit the interferences between the three sensors.

A control algorithm inspired by tropic responses was developed to test the basic behavior of the root apex following chemical stimulation. The movement of the tip following the chemical stimulus was assured by the soft actuation system [26].

A customized graphical user interface (GUI) was developed (Microsoft VB.NET) to configure the robotic root algorithm parameters, to calibrate the sensors, and to acquire and recorded data from the tip.

### 2.3. Sensor Characterization in Solutions and Gels

The sensors were characterized individually, first using solutions containing one analyte. In particular, the response of the pH ISE was tested in 0.1 M Tris-HCl-based solutions with different pH values ranging from 4 to 8 and the response of the pK ISE was tested in 0.1 M Tris-HCl at pH 7.4, containing different KCl concentrations ranging from 10^−^^5^ to 10^−^^1^ M.

All the membrane potentials were normalized by offsetting all the electrode readings to force the measured signal at pH 8 to be 0 mV. The same was implemented for pK membrane potentials, offsetting the values in 10^−^^5^ M of KCl solution to 0 mV. Each curve was obtained by averaging the normalized potential values from three experiments.

Once the sensors in solution were optimized and characterized, a simulated soil using gel was constructed that allowed the generation of pH and potassium concentration gradients. A setup consisting of different Phytagel™ layers was used containing one analyte (pH of 6–8 or [K^+^] of 10^−^^5^–10^−^^1^ M).

For the gel preparation for single-analyte tests, 0.1 M Tris-HCl-based solutions with a fixed pH (pH of 7.4) were prepared with the desired KCl concentration. Subsequently, 0.6% *w*/*v* Phytagel^TM^ powder was added to each solution together with 0.1% *w*/*v* of anhydrous MgSO_4_ and stirred vigorously for a minimum of 20 min. The mixtures were autoclaved for 20 min at 120 °C to achieve complete dissolution of the gel powder. Each solution was then poured in a Petri dish and left to jellify at room temperature. Gel slices were cut and manually applied directly on the sensor’s surface. In a typical experiment, the sensors were preliminarily swelled in the presence of deionized water to activate the ISE membrane. Afterward, the gel sample at the lower concentration was applied for 10 min, and an offset between the sensors was removed by means of the GUI. For gels containing control samples of pH and potassium, the preparation was analogous to the one for the single analytes reported above, but the pH was regulated to the value detailed in Table 1 by adding HCl.

In these first experiments, the chemical sensors’ behavior was analyzed by potentiometry with a commercial portable PalmSens electrochemical interface (PalmSens, Utrecht, The Netherlands) to compare with the integrated potentiostat in the robotic system. The potential difference between the WEs and the RE was measured for different known concentrations of the ions to be detected. The sensitivity of the different sensors was investigated by comparing the slopes obtained from the linear relationship between the logarithm of the ion concentration and the voltage registered with the corresponding ISE.

### 2.4. Artificial Chemotropism Sensing Experimental Protocol

To validate the robotic root’s chemotropic behavior, the sensors were integrated into the robotic root, and the robotic apex was put in contact with a gel-based chemical gradient. The control algorithm was then set to follow decreasing pH and increasing [K^+^]. The protocol used to evaluate the tropic responses from the artificial root consisted of the following steps:

1. Configure the root algorithm.
2. Fix a vertical starting position for the root (gravitropism with no chemical stimuli applied).
3. Apply the gel with a specific concentration on the three arrays of sensors in different directions.
4. Remove offsets by means of a software interface after some minutes.
5. Apply a different gel condition on sensor #2.
6. After stabilization, apply the gel condition used in step 5 on sensor #1.
7. After stabilization, apply the gel condition used in step 5 on sensor #3.

During the change of the gel sample, the control algorithm was turned off to avoid noise influence on the measurements.

## 3. Results and Discussion

### 3.1. Sensor Optimization and Characterization

Soil pH monitoring is of high importance as the pH strongly influences the availability of nutrients and the presence of microorganisms and plants. Most plants prefer a pH range from 5.5 to 7.5, but some species prefer more acidic or alkaline soils. Potassium, together with nitrogen and phosphorous, is one of the most important nutrients in agriculture, and all the three parameters are prime ingredients in almost all fertilizers. Kim et al. [27,28,29] showed that a sensitivity of K^+^ ISE membranes in the range of 10^−^^4^ to 10^−^^1^ in soil extracting solutions was enough to measure the typical range in soil potassium at which additional K^+^ fertilizer is recommended. For this reason, a pH range of 4 to 8 and a potassium concentration range from 10^−^^5^ to 10^−^^1^ M were selected for the calibration curve of the pH and pK sensors devoted to soil analysis.

**Figure 1:**
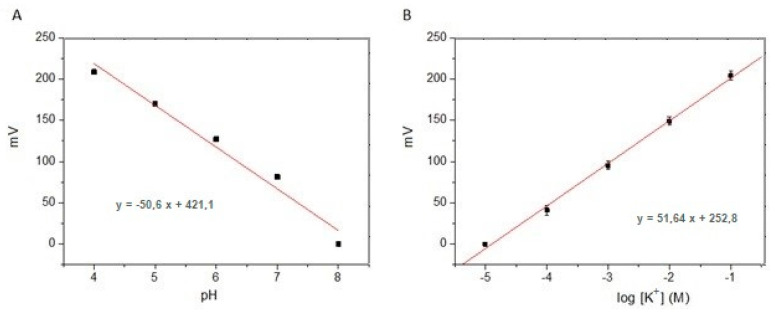
Potentiometric response (n = 5) of (**A**) pH sensor in 0.1 M Tris-HCl solutions of different pH values and (**B**) K^+^ sensor in 0.1 M Tris-HCl solution (pH 7.4) of different KCl concentrations.

The performance of the sensors was first optimized and characterized in solutions at specified pH and potassium concentrations. As shown in Figure 1, the potential values registered with the pH- and pK-tested membranes inside solution were linearly proportional to the logarithm of the H^+^ or K^+^ concentration, respectively. The sensitivity was 52 mV/dec for [K^+^] and 51 mV/dec for pH. The slight deviation from Nernstian behavior could be due to the use of the Ag/AgCl planar solid-state RE, as it is known that stability of these type of electrodes is not as high as that of liquid-junction electrodes [30,31].

**Figure 2:**
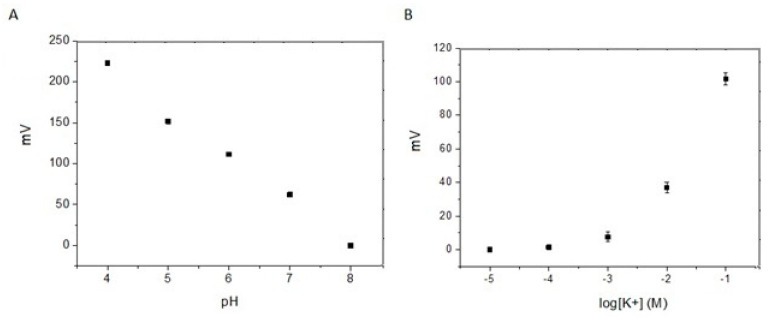
Potentiometric response (n = 5) of (**A**) a pH sensor in gels made with Phytagel^TM^ powder and 0.1 M Tris·HCl solutions of different pH values and (**B**) a K^+^ sensor in gels made with Phytagel^TM^ powder and 0.1 M Tris·HCl solution (pH 7.4) of different KCl concentrations.

Once the sensors were optimized on controlled-concentration solutions of pH and potassium, the sensors were characterized by measurement on simulated soil using Phytagel^TM^. Figure 2 shows the response of the pH and pK membranes inside Phytagel^TM^ for different pH values and KCl concentrations. Since Phytagel^TM^ itself contains some potassium (1.7% according to manufacturer specifications), the lower measured concentration of potassium (10^−^^5^–10^−^^4^) did not produce an increase in the sensor response, as this potassium concentration is already present in the Phytagel^TM^ background signal. Therefore, the differences in [K^+^] observed in gels prepared from solutions with concentrations lower than 10^−^^3^ M KCl were almost negligible, and we lost the linearity of the calibration curve up to 10^−^^3^. However, clear differences were observed above 10^−^^3^ M KCl and under different pH concentrations.

### 3.2. Sensor Integration in the Robotic Tip

Several difficulties were faced in the integration of the array of sensors in the artificial robotic root tip. There were a few specific challenges for the electronic system: firstly, the high internal impedance of the ISE sensors; secondly, during a reading task from all electrodes, the short distance (8 mm) between WEs meant that parasite currents between them could skew output values; and finally, there were challenges in reading from three couples of electrodes at the same time. To overcome these issues, a micro-potentiometer was developed for the root tip electronics, which integrated a microcontroller unit and a communication unit. For each electrode, a high-input-impedance amplifier was used, and for the RE, three individual reference voltages of the same value were used and supplied with a high-value resistance (10 MΩ) to limit the interferences between the three sensors.

To test the basic behavior of the root apex due to chemical stimulations, a control algorithm inspired by tropic responses was developed and t he movement of the tip was assured by the soft actuation system [26].

**Figure 3:**
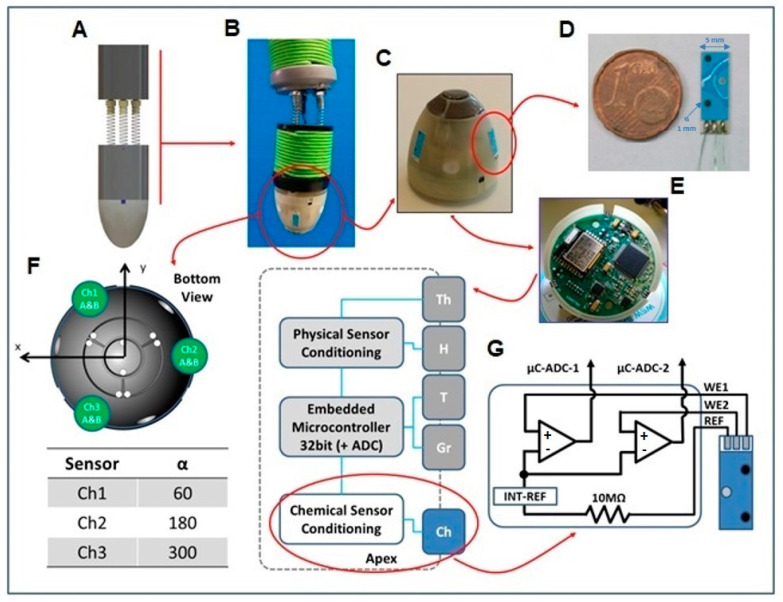
Scheme of the robotic biomimetic root with sensors integrated. (**A**) Soft-bending mechanism design. (**B**) Prototype. (**C**) Root tip prototype with integrated chemical sensors. (**D**) ISE prototype. (**E**) Micro-potentiometer integrated in the root tip. (**F**) Sensors’ position with respect to the x–y axis of the embedded accelerometer. (**G**) Tip architecture with chemical sensor’s front-end schematization for each of the three sensor arrays.

Gravity is a constant stimulus in the environment that the plant perceives continuously; therefore, chemotropism occurs in addition to gravitropism, and the final behavior of the root is the result of the unavoidable influence of both types of stimuli: chemical species and gravity. For this reason, both sensors were integrated in the robotic root. The algorithm calculates the direction of the applied stimuli and follows the resulting stimulus until it is completely compensated by the gravity. It is possible to write, for each species, the following equations:

(1) $${Ch}_{x}=\left|{Ch}_{1}-{Ch}_{K}\right|\cdot \cos\alpha_{{Ch}_{1}}+\;\left|{Ch}_{2}-{Ch}_{K}\right|\cdot \cos\alpha_{{Ch}_{2}}+\left|{Ch}_{3}-{Ch}_{K}\right|\cdot \cos\alpha_{{Ch}_{3}}$$

(2) $${Ch}_{y}=\left|{Ch}_{1}-{Ch}_{K}\right|\cdot \sin\alpha_{{Ch}_{1}}+\left|{Ch}_{2}-{Ch}_{K}\right|\cdot \sin\alpha_{{\mathrm{Ch}}_{2}}+\left|{Ch}_{3}-{Ch}_{K}\right|\cdot \sin\alpha_{{Ch}_{3}}$$

(3) $$r_{Ch}=\sqrt{{Ch}_{x}^{2}+{Ch}_{y}^{2}}$$

(4) $$\theta_{Ch}=\tan^{-1}\left(\frac{{Ch}_{y}}{{Ch}_{x}}\right)$$

where ${Ch}_{i}$ are the measurements supplied by the three chemical sensors (pH or pK). $\alpha_{{Ch}_{i}}$ are the angles of each sensor with the *x*-axes (defined in Figure 3). ${Ch}_{K}$ is the ideal value for the species, and the “distance” of each sensor with respect to this value is measured. $\theta_{Ch}$ is the angle with respect to the *x*-axes (of the accelerometer) of the species resultant vector. $r_{Ch}$ is the module of the chemical species resultant vector. For the gravity sensor, it is possible to obtain $\theta_{G}$, which is the angle with respect to the *x*-axes of the projection of the gravity vector on the *x*/*y* plane.

The intensity of the stimulus ($r_{G}$) is as follows:

(5) $$r_{G}=\sin\varphi_{G}$$

where $\varphi_{G}$, is the angle between the tip direction and the gravity vector.

All the stimuli combined give the direction and then the speed of the motors necessary to obtain the bending. Figure 3 shows the design of the bioinspired robotic root with the integrated sensors and electronics.

### 3.3. Characterization of Sensors’ Integration in the Robotic Root

Three sensor arrays were designed and fabricated in a way that allowed them to come into contact with the selected medium and to be easily integrated in the robotic apex of an artificial root that imitates the differential bending for growth toward the optimal conditions for natural roots [24].

The experiments with the robotic tip for chemotropism analysis were performed using a pH range from 6 to 8 and a potassium concentration [K^+^] of 10^−^^3^ to 10^−^^1^ M. This pH range was selected because it is common in many soils. Furthermore, the use of highly acidic conditions affects the consistency of Phytogel^TM^, making it too soft. In the case of K^+^, this range of concentration is the most common in soil and also is the concentration range in which the presence of potassium in the Phytagel^TM^ recipe does not affect the sensitivity of the sensor.

The sensors were tested with different K^+^ and pH concentration combinations, as reported for gels G1-9 (Table 1). The analysis of the potentiometric response of the integrated sensor in the modified gel was performed with a commercial potentiostat, followed by our specific readout instrumentation to evaluate both sensors and electronics, confirming their robustness in a soil-like environment. In these characterization experiments, gels in slices with a thickness of a few millimeters and a size about the same of the sensor were applied directly on the sensor’s array to guarantee good contact between the gel and the sensor’s electrodes.

These experiments started with lower-concentration gels (G1), applied for 10 min, and an offset between the sensors was obtained by means of the GUI. Gels with increasing [K^+^] (G2–3) were applied at the same pH, and then, the experiment was repeated with the other pH (G4–9). This procedure was repeated for four sensorized tips to obtain accurate reproducibility.

**Figure 4:**
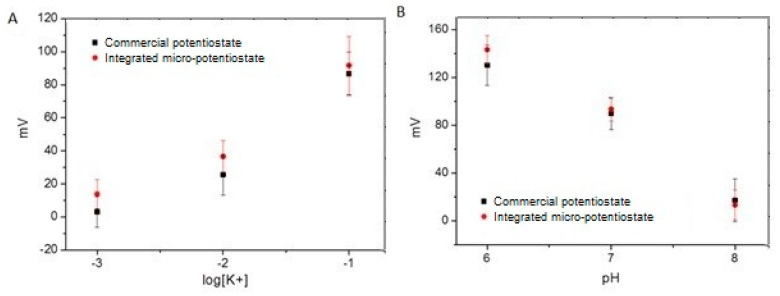
Comparison between pH (**A**) and K^+^ (**B**) measurements performed on Phytagel^TM^ G1–9 with commercial potentiostat (squared black data) and with the Plantoid robot (circled red data) n = 4.

Data acquisition with the robotic root tip was compared with the same analysis performed by a commercial potentiostat. A good reproducibility was verified with the developed and integrated readout. The graphs in Figure 4 show the pH sensor and the K^+^ sensors in contact with G1–9, measured both with the PalmSens device (squared black data) and with the robotic root (circled red data). The pH measurements show very good reproducibility; meanwhile, the K^+^ measurements show about 10 mV of offset. Both results demonstrated good capabilities to measure a gradient of pH/K^+^ in the gel.

The cross-sensitivity was studied and indicated a limited influence of pH sensing on K^+^ sensing. Also, in nature, a plant’s chemosensitive system displays a cross-sensitivity to pH and K^+^, because soil pH can influence potassium perception by plant roots [2].

The pH sensors showed very good linearity, close to the Nernst law, and low cross-sensitivity to the [K^+^] variation. A slight deviation from the ideal behavior was registered for G3. This can be observed in Figure 4B, in which the results for G3 at pH 8 contribute to increasing the data dispersion for log[K^+^] equal to −1.

Also, the potassium sensors showed good linearity even when the cross-sensitivity to pH variation was higher; in particular, only the G9 potassium sensors (low pH (6) and high [K^+^] (10^−^^1^ M)) showed reduced accuracy.

Comparing the sensors’ performance under laboratory measurement conditions with commercial equipment and connectors shown in Figure 2 and the performance of the same sensors integrated into the robotic platform (Figure 4), a greater deviation in the results was observed in the sensors integrated into the robotic system. This higher dispersion can probably be produced by the variability in the contacts of the electrodes with the miniaturized electronic circuit of the robotic system, made by hand soldering, which could generate variation from platform to platform. A shift in the signal of the integrated circuit in the robot with respect to the commercial potentiostat was also observed, which may come from the difference in quality between the integrated circuit and the commercial one, which is more isolated and protected from external electrical noise. The performance of the sensor integrated in the robot was satisfactory and allowed for the implementation of a response of the robot based on a given concentration. The next experiment allowed us to combine the performance of the sensor response to generate a stimulus for the robot to move in a specific direction based on the increase/decrease in the pH and/or pK concentration following the guidelines set in its program. So, the next step was to recognize the chemical gradients as robotic stimuli.

**Figure 5:**
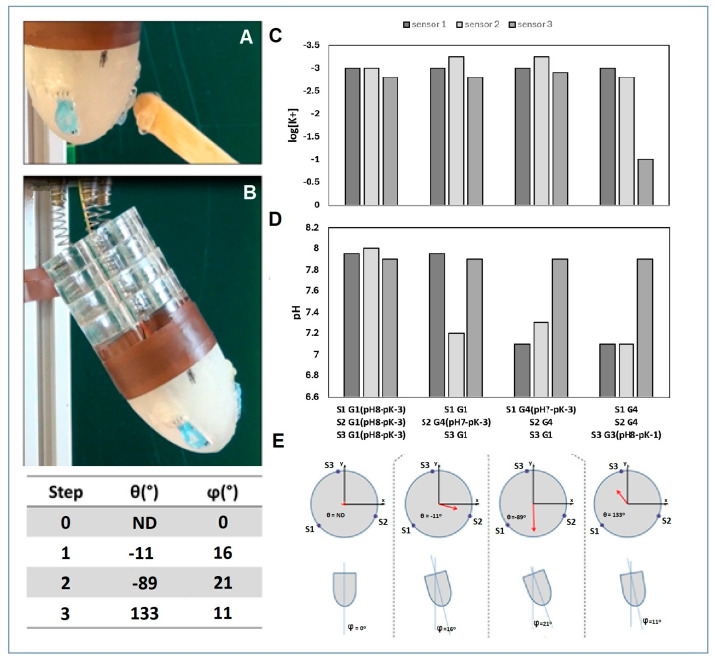
Root-based robotic sensor’s response to different pH and potassium stimuli. (**A**) Gel application on the ISE sensors. (**B**) Bending of the robotic root used for tropism experiments. (**C**) K^+^ sensors’ response from the arrays on the robot tip under different stimuli. (**D**) Data acquired from the pH sensors from the arrays on the robot tip under different stimuli. (**E**) Tip angle extracted by the accelerometer data. These angles are shown in the tip by vectors: *θ* is the rotation of the tip with respect to the *x*-axes, and *φ* is the bending represented as the module of the vector.

To validate this behavior, three sensor arrays were assembled in the robotic root. The robotic apex, in which the sensors were placed, was put in contact with a gel chemical gradient combining a controlled concentration of pH and pK, and the robot’s ability to move based on the response detected by the sensors was investigated. The results are summarized in Figure 5, in which the root apex’s position after the gel application and the sensors’ responses are depicted.

The experiment started with all the sensors in the robotic apex inside the G1 gel (pH 8 and pK 10^−3^ M) and with a rotation angle of 0 from the robotic apex (Figure 5A). Figure 5C,D, respectively, depict the response of the three arrays containing the pK and pH sensors to different gel conditions. The voltage responses of the three arrays were extrapolated onto the calibration curve in Figure 2 to graphically represent the pK and pH values. The graphs in Figure 5E show the deflection of the robot relative to the position of the sensors. In the first step of the experiment, G4 (pH 7 and pK 10^−^^3^ M) was applied on sensor 2. The sensor detected an increase in protons due to the lower pH, with a consequent increase in mV. The robot detects an increase in voltage at a specific position of its apex and the program commands the robot apex to bend in the direction of sensor 2, which is equivalent to an angle of *θ* −11 and *φ* 16. The third step of the experiment was to place G4 on sensor 1, which also generates a decrease in pH but the same decrease in mV as in sensor 1. The program then directs the root apex to bend between sensors 1 and 2 (*θ* −89 and *φ* 21), which are the areas of the root in which a more suitable pH for the plantoid is detected. In the last step of the experiment, G3 (pH 8 and pK 10^−^^1^ M) was applied to sensor 3, in which the pH is the same as the initial one, but the potassium concentration is increased. As no differences in the pH response were observed but an increase due to the potassium sensor was detected, the root tip bent a specific percentage determined by the program due to an increase in mV of the potassium sensor in the direction of sensor 3, compensating for and overcoming the other stimulus (*θ* 133 and *φ* 11) (Figure 5D).

The successful results of this experiment prove the good correlation of the robot’s movements with the concentrations detected by the sensor arrays integrated in the robot apex, generating an autonomous artificial intelligence that imitates the chemotropism of plants.

## 4. Conclusions

Sensing via functionalized chemical sensors has traditionally been neglected in robotics due to the technical challenges of its implementation in these systems. However, the integration of chemical sensing capabilities into robotics adds a new dimension of functional capabilities to this technology. Nature can give us many examples of detection to apply a decision, basic in the survival of animals and plants, such as the ability to detect food. Thus, the combination of sensing with artificial intelligence and robotics offers new, added capabilities for environmental detection, as well as multiple possibilities in different areas, such as the proposal in this work for the detection of nutrients in soil, based on the capabilities of the roots of the plants to grow toward the maximum nutrient concentration.

In this work, a miniaturized array integrated in a robotic tip inspired by plant-root chemotropism was developed. The system is able to measure [K^+^] and pH three-dimensionally in a moist, soil-like environment (Phytagel^TM^) and move in a preferred direction in presence of chemical gradient stimuli in a value range compatible with real-soil scenarios. The sensory platform is composed of three arrays containing planar potentiometric ISE sensors for K^+^ and pH detection. The sensors demonstrated good linearity close to the Nernst law and a low cross-sensitivity to similar ions.

The integration of robotic platforms and miniaturized sensors shows a correlation similar to the reading of commercial potentiostats and a correct response of the robot considering the stimuli received at the sensors. This has enormous potential for autonomous environmental monitoring and in situ soil exploration using intelligent robotic systems.

## Figures and Tables

**Table 1:** List of the prepared gel samples used to test the chemical sensors.

Sample	Log [K^+^] (M)	pH
**G1**	**−3**	**8**
**G2**	**−2**	**8**
**G3**	**−1**	**8**
**G4**	**−3**	**7**
**G5**	**−2**	**7**
**G6**	**−1**	**7**
**G7**	**−3**	**6**
**G8**	**−2**	**6**
**G9**	**−1**	**6**

## Data Availability

The original contributions presented in the study are included in the article material, further inquiries can be directed to the corresponding author.
